# Association between Genetic Variants in *DUSP15*, *CNTNAP2*, and *PCDHA* Genes and Risk of Childhood Autism Spectrum Disorder

**DOI:** 10.1155/2021/4150926

**Published:** 2021-06-28

**Authors:** Fang Fang, Minxia Ge, Jun Liu, Zengyu Zhang, Hong Yu, Shuilong Zhu, Liwei Xu, Lina Shao

**Affiliations:** ^1^Department of Clinical Laboratory, The Third People's Hospital of Xiaoshan District, Hangzhou, China; ^2^Central Laboratory, Department of Clinical Laboratory, Zhejiang Xiaoshan Hospital, Hangzhou, China; ^3^Department of Pediatrics, Xiaoshan First Affiliated Hospital of Hangzhou Normal University, Hangzhou, China; ^4^Department of Clinical Psychology, Xiaoshan First Affiliated Hospital of Hangzhou Normal University, Hangzhou, China

## Abstract

**Objective:**

Genetic factors play an important role in the development of autism spectrum disorder (ASD). This case-control study was to determine the association between childhood ASD and single nucleotide polymorphisms (SNPs) rs3746599 in the *DUSP15* gene, rs7794745 in the *CNTNAP2* gene, and rs251379 in the *PCDHA* gene in a Chinese Han population.

**Methods:**

Genotypes of SNPs were examined in DNA extracted from blood cells from 201 children with ASD and 200 healthy controls. The Children Autism Rating Scale (CARS) was applied to evaluate the severity of the disease and language impairment. The relationship between SNPs and the risk of ASD or the severity of the disease was determined by logistic regression and one-way ANOVA.

**Results:**

The genotype G/G of rs3746599 in the *DUSP15* gene was significantly associated with a decreased risk of ASD (odds ratio (OR) = 0.65, 95% confidence interval (CI): 0.42-0.99, *P* = 0.0449). The T allele of rs7794745 in the *CNTNAP2* gene was associated with an increased risk of ASD (OR = 1.34, 95% CI: 1.01-1.77, *P* = 0.0435). The SNP rs251379 was not associated with ASD. Though none of the SNPs examined were associated with ASD severity, rs7794745 was associated with severity of language impairment.

**Conclusions:**

Our findings suggest that both rs3746599 in the *DUSP15* gene and rs7794745 in the *CNTNAP2* gene are associated with risk of childhood ASD, and rs7794745 is also related to the severity of language impairment in autistic children from a Chinese Han population.

## 1. Background

Autism spectrum disorder (ASD) is a neurodevelopmental disorder that presents with social and communicative deficits and repetitive behaviors. With increased attention and improved diagnoses, the prevalence of the disease has been increasing in China [1]. The wide prevalence of this disease brings huge economic burdens to families and society. Early diagnosis and intervention are essential to improve the efficacy of treatment. Though the etiology of the disease has not been fully elucidated, genetic factors have been implicated as important risk factors in the pathogenesis of ASD [2, 3]. One of the most common types of genetic variants, single nucleotide polymorphisms (SNPs), in certain genes may impact the susceptibility to ASD. Identification and validation of these ASD-related SNPs will assist in the development of early diagnoses and more efficient treatments.

Dual-specificity phosphatase 15 (DUSP15) is a member of the family of dual-specificity phosphatases possessing the ability to dephosphorylate both phosphoserine/threonine and phosphotyrosine residues. The *DUSP15* gene was shown to regulate myelination in oligodendrocytes and their differentiation and development [4]. Myelination control and axon support by oligodendrocytes are essential for the normal functions of complex neural cellular networks [5]. Pathology of oligodendrocytes has been correlated with ASD. Patients with ASD were found to have fewer oligodendrocytes in the amygdala compared to typically developing controls [6]. In addition, the intronic SNP rs3746599 in the *DUSP15* gene has been identified as a risk factor for childhood ASD [7].

The *CNTNAP2* gene product is a member of the neurexin superfamily of proteins that are highly expressed throughout the brain and spinal cord and facilitate cell–cell interactions in the nervous system [8]. By interacting with transient axonal protein 1, the CNTNAP2 protein at the juxtaparanodal region stimulates myelinating glial cells to organize ion channels in the underlying axonal membrane [9]. Independent studies have demonstrated a positive correlation between mutations in *CNTNAP2* and susceptibility for ASD [10–13]. Transgenic mice lacking *Cntnap2* display striking similarity to the core deficits in behavioral and cognitive functions observed in patients with ASD [14]. The intronic SNP rs7794745 in the *CNTNAP2* gene was reported to be a risk factor for ASD in some studies [15–17], but not others [18, 19].

The protocadherins (PCDH) are the cadherin superfamily of calcium-dependent cell adhesion molecules which include the nonclustered genes and the *α*, *β*, and *γ* clustered genes [20, 21]. The *PCDHα* (*PCDHA*) is composed of 15 large, variable region exons arrayed in tandem and a constant region composed of 3 exons [21]. The *PCDHA* and other *PCDHs* mediate homophilic matching between different cell types for the formation and function of complex neural networks [22]. The PCDHA protein is localized at the specific synaptic junctions and thus plays an important role in specific synaptic connections [20]. Impaired neuronal connectivity due to altered synapses has been attributed to cognitive deficits [23]. This protein is also important for appropriate innervation of target brain areas by serotonergic projections [24]. Abnormal serotonin signaling has been associated with the development of ASD [25, 26]. Furthermore, the SNP rs251379, localized in the introns of the *PCDHA1-12* genes, has been reported to be correlated with the risk of ASD [27]. Taken together, these findings imply that the PCDHA is a possible susceptibility gene for ASD.

Previous studies suggest that *DUSP15*, *CNTNAP2*, and *PCDHA* are important in maintaining the functional connectome of the brain and optimal information processing in complex neural networks. It is noted that the aforementioned SNPs in these genes have only been individually studied in different populations. Due to the wide ranges of disease presentations caused by varied genetic and environmental factors, findings for ASD-related SNPs need to be corroborated in individual ethnic populations. The objective of this study was to determine the relationship between childhood ASD and the SNPs rs3746599 in *DUSP15*, rs7794745 in *CNTNAP2*, and rs251379 in *PCDHA* in a Chinese Han population. Related SNPs may serve as candidate biomarkers for the diagnosis of ASD in the future studies.

## 2. Patients and Methods

A total of 201 autistic children and 200 age- and gender- matched healthy children were enrolled in this study from 2012 to 2016 [28–30]. The severity of ASD was evaluated using the Children Autism Rating Scale (CARS). There are 14 domains to evaluate individual behaviors and a 15th domain for general impressions in CARS. Each domain is scored from one to four. A higher score indicates a more severe symptom of the domain. Autistic children with total scores < 36 were classified as mild/moderate, and total scores ≥ 36 were classified as severe. The language communication domain scores in CARS were used to evaluate the severity of language impairment in autistic children. This study was approved by the Medical Ethics Committee of Zhejiang Xiaoshan Hospital. Informed consent was obtained from parents or guardians of all children.

Genotypes were analyzed in DNA extracted from blood cells using a TaqMan probe-based approach. The probes were designed and synthesized by Applied Biosystems (Beijing, China). Real-time PCR was conducted following the manufacturer's protocol, described previously [31].

For statistical analysis of all SNP frequencies, the Hardy-Weinberg equilibrium was examined using the *χ*^2^ test. Logistic regression analysis was applied to examine the association between SNPs and the risk of childhood ASD or severity of the disease. Odds ratios (ORs) and 95% confidence intervals (CIs) were calculated. One-way ANOVA was applied to analyze the difference in language communication domain scores among the SNPs of interest. Group-wise comparisons were made using the Tukey test. Statistical analyses were performed using the SAS 9.3 software (SAS Institute Inc., Cary, NC). A *P* value < 0.05 was considered to be significant.

## 3. Results

Genotype distributions of rs3746599 in *DUSP15*, rs7794745 in *CNTNAP2*, and rs251379 in *PCDHA* were all consistent with the Hardy-Weinberg equilibrium in both case and control groups (Table 1).

Results from logistic regression analysis showed that the G/G genotype of rs3746599 in *DUSP15* (OR = 0.65, 95% confidence interval (CI) = 0.42–0.99, *P* = 0.0449) was significantly associated with decreased risk of childhood ASD. There was no significant difference in allele frequency of rs3746599 in autistic children compared to healthy controls (Table 2). The T/T genotype of rs7794745 in *CNTNAP2* was marginally associated with an increased risk of ASD (OR = 1.79, 95%CI = 0.99–3.23, *P* = 0.0542). The T allele was significantly associated with an increased risk of ASD (OR = 1.34, 95%CI = 1.01–1.77, *P* = 0.0435) (Table 2). Neither genotype nor allele distributions of rs251379 in *PCDHA* were significantly associated with risk of childhood ASD (Table 2).

The relationship between these SNPs and childhood ASD was further analyzed using dominant and recessive models of inheritance for rs3746599 in *DUSP15*, rs7794745 in *CNTNAP2*, and rs251379 in *PCDHA*. No significant association between these SNPs and risk of ASD was observed in any of these models (Table 3).

Children affected with ASD were classified into mild/moderate and severe groups based on CARS scores. There were 122 children with mild-to-moderate ASD and 79 with severe ASD. Logistic regression analysis showed that there was no significant association between the presence of these SNPs and the severity of childhood ASD (Table 4).

The association between the SNPs and the severity of language impairment was further examined in autistic children. Our results indicated that the genotype A/A was associated with a significantly lower language communication domain score compared to the T/T or T/A genotypes of rs7794745 in *CNTNAP2*. There was no significant difference in language communication domain score among genotypes of rs3746599 in *DUSP15* or rs251379 in *PCDHA* (Table 5).

## 4. Discussion

ASD is a multifactorial disease caused by both genetic and environmental factors. Our current study determined the association between three SNPs and childhood ASD in a Chinese Han population. The results showed that the SNP rs3746599 in the *DUSP15* gene and rs7794745 in the *CNTNAP2* gene, but not rs251379 in *PCDHA*, were risk factors for childhood ASD. Although none of the SNPs were associated with severity of the disease, rs7794745 in *CNTNAP2* was significantly associated with severity of language impairment.

As a key regulator for oligodendrocyte differentiation and myelin production, the *DUSP15* gene plays an important role in the development and function of the nervous system [32]. The rs3746599 SNP in the DUSP15 gene was predicted to be within the conserved transcription factor binding site and thus influence the binding ability of transcription factors and, consequentially. gene expression [33]. A previous study determined an association between the *DUSP15* SNP rs3746599 and childhood ASD in a Chinese Han population. A total of 255 autistic children and 427 healthy controls were enrolled in the case-control study. SNP rs3746599 genotypes were examined by Sanger sequencing. Their results showed that SNP rs3746599 was significantly associated with childhood ASD under allelic, additive, and dominant models [7]. Using the same ethnic population from a different region in China, our data also showed that SNP rs3746599 in *DUSP15* was significantly associated with ASD. Taken together, results from both studies indicate that *DUSP15* is a susceptibility gene for ASD and that SNP rs3746599 is a potential marker that might be used in screening for ASD in this population.

Several groups have studied the association between rs7794745 in the *CNTNAP2* gene and the risk of ASD. An Iranian case-control study analyzed rs7794745 genotypes and frequencies in a total of 200 autistic patients and 260 healthy individuals. The genotype frequencies of AA, AT, and TT were 35.3%, 50.7%, and 13.8% in controls and 32%, 68%, and 0% in autistic patients, respectively. SNP rs7794745 in *CNTNAP2* was significantly associated with ASD in this population [15]. A significant association between the T allele of rs7794745 and the increased risk of high functioning ASD was reported in the case-control study from a European group [16]. A Brazilian group examined *CNTNAP2* rs7794745 and rs2710102 in 210 patients with ASD and 200 healthy individuals. Their results revealed a significantly higher frequency of the TT allele of rs7794745 in ASD patients compared to controls [17]. Consistent with this finding, this study found a significant association between the T allele of rs7794745 and increased risk of ASD in a Chinese Han population in this study. Another study in the same Chinese Han population showed a significant correlation between ASD and haplotype T-A (rs7794745-rs10500171, *P* = 0.011) or haplotype A-T-A (rs10244837-rs7794745-rs10500171) of the *CNTNAP2* gene [34]. However, several other studies reported no significant association between rs7794745 and the risk of ASD [18, 19].

The role of the *PCDHA* SNP rs251379 in the development of ASD was also evaluated in this study. No significant association between rs251379 and ASD was observed. In contrast to this finding, a previous study examined the genetic association of PCDHA with ASD in a cohort of 841 families that included a total of 3211 individuals. Among 14 *PCDHA* SNPs examined, rs251379 was one of the five SNPs significantly associated with ASD [27]. This result suggests more study is needed to validate rs251379 in the *PCDHA* gene as a risk factor for ASD in a Chinese population.

Language impairment is a common sign of ASD. The severity of language impairment was based on the scores of the language communication domain in the CARS. This study determined the correlation between three SNPs and severity of language impairment in children with ASD. Our results revealed that only rs7794745, but not rs3746599 or rs251379, was significantly associated with severity of language impairment. Several independent studies reported the effects of the rs7794745 SNP in the *CNTNAP2* gene on the brain activities of vocal communication and language processing in healthy individuals [35–38]. Homozygous risk allele of rs7794745 was associated with significant reductions in grey and white matter volume and fractional anisotropy in several regions [39]. All these findings suggest that the impact of rs7794745 on brain structure and brain activities during language processing may contribute to the severity of language impairment observed in this study. More studies are needed to elucidate the mechanisms accounting for the effect of rs7794745 on language impairment in patients with ASD.

## 5. Conclusions

Our study finds that the *DUSP15* rs3746599 and *CNTNAP2* rs7794745, but not *PCDHA* rs251379, are associated with the risk of childhood ASD in a Chinese Han population. In addition, rs7794745 in *CNTNAP2* was significantly associated with severity of language impairment in autistic children.

## Figures and Tables

**Table 1:** *P* values of Hardy-Weinberg equilibrium tests of SNP in case and control groups.

	Cases	Controls
rs3746599	0.0568	0.3524
rs7794745	0.7110	0.8801
rs251379	0.2792	0.3086

**Table 2:** Correlation between SNP genotypes and allele frequencies and childhood ASD.

SNPs	Genotype/allele	Cases *n* (%)	Controls *n* (%)	OR (95% CI)	*P* value
rs3746599	A/A	89 (44.3)	71 (36.2)	1	
	A/G	80 (39.8)	99 (50.5)	0.98 (0.54-1.80)	0.9526
	G/G	32 (15.9)	26 (13.3)	0.65 (0.42-0.99)	0.0449
	A	258 (64.2)	241 (61.5)	1	
	G	144 (35.8)	151 (38.5)	0.89 (0.67-1.19)	0.4314

rs7794745	A/A	60 (29.9)	77 (38.5)	1	
	A/T	102 (50.8)	95 (47.5)	1.38 (0.89-2.14)	0.1516
	T/T	39 (19.4)	28 (14.0)	1.79 (0.99-3.23)	0.0542
	A	222 (55.7)	249 (65.3)	1	
	T	180 (44.3)	151 (37.7)	1.34 (1.01-1.77)	0.0435

rs251379	A/A	45 (22.4)	40 (20.1)	1	
	A/G	92 (31.8)	90 (45.2)	0.83 (0.48-1.42)	0.4880
	G/G	64 (45.8)	68 (34.7)	0.91 (0.54-1.52)	0.7161
	A	182 (45.3)	170 (42.7)	1	
	G	220 (54.7)	228 (57.3)	0.90 (0.68-1.19)	0.4660

OR: odds ratio; CI: confidence interval.

**Table 3:** Genotype distributions and corresponding risk predictions for ASD under genetic models of inheritance.

SNP	Model	Genotype	Cases *n* (%)	Controls *n* (%)	OR (95% CI)	*P* value
rs3746599	Dominant	A/A	89 (44.3)	71 (35.5)		
		A/G+G/G	112 (55.7)	129 (64.5)	0.69 (0.46-1.04)	0.0731
	Recessive	A/A+A/G	169 (84.1)	174 (87.0)		
		G/G	32 (15.9)	26 (13.0)	1.27 (0.72-2.22)	0.4065

rs7794745	Dominant	A/A	60 (29.9)	77 (38.5)		
		A/T+T/T	141 (70.2)	123 (61.5)	1.47 (0.97-12.23)	0.0684
	Recessive	A/A+A/T	162 (80.6)	172 (86.0)		
		T/T	39 (19.4)	28 (14.0)	1.48 (0.87-2.51)	0.1485

rs251379	Dominant	G/G	64 (31.8)	69 (34.5)		
		A/A+A/G	137 (68.2)	131 (65.5)	1.12 (0.74-1.69)	0.5719
	Recessive	A/G+G/G	156 (77.6)	160 (80.0)		
		A/A	45 (22.4)	40 (20.0)	1.15 (0.71-1.85)	0.5591

OR: odds ratio; CI: confidence interval.

**Table 4:** Correlation between SNP genotypes and allele frequencies and severity of childhood ASD.

	Genotype/allele	Mild/moderate *N* (%)	Severe *N* (%)	OR (95% CI)	*P* value
rs3746599	A/A	50 (41.0)	39 (49.4)	1	
	A/G	54 (44.3)	26 (32.9)	0.62 (0.33-1.16)	0.3210
	G/G	18 (14.7)	14 (17.7)	1.00 (0.44-2.25)	0.9945
	A	154 (63.1)	104 (65.8)	1	
	G	90 (36.9)	54 (34.2)	0.89 (0.58-1.35)	0.5803

rs7794745	A/A	35 (29.5)	25 (31.7)	1	
	A/T	60 (49.2)	42 (53.2)	0.98 (0.51-1.87)	0.9512
	T/T	27 (21.3)	12 (15.2)	0.62 (0.27-1.46)	0.2751
	A	130 (54.1)	92 (58.2)	1	
	T	114 (45.9)	66 (41.8)	0.82 (0.55-1.23)	0.3300

rs251379	A/A	25 (20.5)	20 (25.3)	1	
	A/G	61 (50.0)	31 (39.2)	0.64 (0.31-1.32)	0.2230
	G/G	36 (29.5)	28 (35.4)	0.97 (0.45-2.10)	0.9427
	A	111 (45.5)	71 (44.9)	1	
	G	133 (54.5)	87 (55.1)	1.02 (0.68-1.53)	0.9131

OR: odds ratio; CI: confidence interval.

**Table 5:** Language communication domain scores among genotype groups in autistic children.

SNP	Genotype	Score (mean ± SD)^∗^	*P* value
rs3746599	A/A	2.6 ± 0.8^a^	
	A/G	2.4 ± 0.8^a^	
	G/G	2.3 ± 0.8^a^	0.3119

rs7794745	A/A	2.2 ± 0.7^a^	
	A/T	2.5 ± 0.7^b^	
	T/T	2.7 ± 0.9^b^	0.0061

rs251379	A/A	2.6 ± 0.8^a^	
	A/G	2.5 ± 0.7^a^	
	G/G	2.4 ± 0.8^a^	0.4174

^∗^Different letter indicates significant difference among the genotypes of the same SNP group.

## Data Availability

The datasets used during the current study are available from the corresponding author on reasonable request.
